# The promising potential of intra‐articular losartan combined with scaffold implantation for osteochondral repair: a comparative study in a rabbit model against osteochondral autograft transplantation

**DOI:** 10.1002/jeo2.70451

**Published:** 2025-10-09

**Authors:** Mesut Ok, Mahmud Aydin, Serkan Surucu, Halide Nur Urer, Mehmet Halis Cerci, Murat Yilmaz, Mesut Sonmez, Mahir Mahirogullari

**Affiliations:** ^1^ Department of Orthopedics and Traumatology Haseki Training and Research Hospital Istanbul Turkey; ^2^ Department of Orthopedics and Traumatology Sisli Memorial Hospital Istanbul Turkey; ^3^ Department of Orthopaedics and Rehabilitation Yale University New Haven Connecticut USA; ^4^ Department of Pathology University of Health Sciences, Hamidiye Faculty of Medicine Istanbul Turkey; ^5^ Department of Orthopedics and Traumatology Prof. Dr. Cemil Tascioglu Training and Research Hospital Istanbul Turkey

**Keywords:** cartilage repair, losartan, osteochondral autograft, osteochondral defect, scaffold

## Abstract

**Purpose:**

This study aimed to compare the radiological, macroscopic, biomechanical and histological outcomes of osteochondral autograft transplantation (OAT) and a combination of intralesional scaffold with intra‐articular losartan injection in a rabbit model of osteochondral injury.

**Methods:**

Twelve rabbits received bilateral osteochondral defects (4 × 4 mm) in the trochlear region of both knees and were divided into two groups. Group A received scaffold implantation with intra‐articular losartan (1 mg per knee, injected in 0.1 mL of solution), while Group B underwent OAT with re‐implantation of the original plug. After 6 months, bilateral knee magnetic resonance imaging (MRI), macroscopic, histological and biomechanical assessments were performed.

**Results:**

Macroscopic examination demonstrated that the defect areas were filled and integrated with the surrounding cartilage. MRI evaluations revealed complete closure of the osteochondral defects. Histological evaluation using Wakitani scores showed no statistically significant difference between the groups (Group A: 3.63 ± 2.13, Group B: 2.63 ± 2.01, *p* = 0.423). Finally, no significant difference was observed in biomechanical load testing between the groups (Group A: 219.2 ± 72.9 N, Group B: 270.8 ± 67.6 N, *p* = 0.149).

**Conclusions:**

The scaffold‐losartan combination may offer a promising, less invasive alternative to traditional OAT for osteochondral defect repair.

**Level of Evidence:**

N/A.

**Abbreviations**
3D: three‐dimensional
EDC: 1‐Ethyl‐3‐(3‐dimethylaminopropyl) carbodiimide
FDA: Food and Drug Administration
G‐CSF: granulocyte colony‐stimulating factor
IV: intravenous
mM: milimolar
MPa: megapascal
MRI: magnetic resonance imaging
MSC: mesenchymal stem cell
NCSS: number cruncher statistical system
NHS: N‐hydroxysuccinimide
OAT: osteochondral autograft transplantation
PBS: phosphate‐buffered saline
TA: acquisition time
TGF‐β: transforming growth factor beta

## INTRODUCTION

Cartilage tissue has a very limited regenerative capacity and its self‐repairing potential is severely restricted [8]. Chondral and osteochondral lesions usually heal by the formation of fibrocartilage, a poorly organised and mechanically less durable tissue than hyaline cartilage [10]. Fibrocartilage is less resistant to mechanical stresses and is prone to deterioration under repetitive loading, frequently leading to the development of osteoarthritis [8, 10].

The primary objectives in the treatment planning of chondral and osteochondral defects are to ensure low cost, minimal morbidity, single‐stage surgery, early mobilisation and long‐term effectiveness. Historically, debridement and microfracture have been among the most well‐established treatment options for cartilage defects. However, the cartilage that forms as a result of these procedures is predominantly fibrocartilage, which is associated with declining success rates over time [9, 22, 26]. In contrast, osteochondral autograft transplantation facilitates the transfer of viable hyaline cartilage with subchondral bone support to the defect site, yielding superior long‐term outcomes compared to microfracture [12]. A significant drawback of osteochondral autografting is donor site morbidity. However, single‐plug OAT is a widely accepted and commonly used solution for small osteochondral defects (e.g., a 9 mm femoral condyle defect successfully filled with a 9 mm OAT plug harvested from the medial trochlear ridge). Thus, the utility of OAT is generally limited to small defects. Moreover, the use of multiple plugs—known as mosaicplasty—has been proposed for larger lesions but has recently declined in popularity due to increased donor site morbidity. As a result, alternative strategies such as fresh osteochondral allografts, acellular osteochondral scaffolds (e.g., MaioRegen), or combined techniques using bone chips, a membrane (e.g., Chondrogide), and bone marrow aspirate concentrate have gained wider acceptance [4, 11, 23]. Nevertheless, a key limitation of osteochondral allograft transplantation remains the need for fresh‐frozen cadaveric tissue and the potential risk of graft‐related complications. Autologous chondrocyte implantation and matrix‐associated autologous chondrocyte implantation are additional therapeutic options, though their disadvantages include high costs and the necessity for a two‐stage procedure. Recently, scaffolds produced using three‐dimensional (3D) printing technology have been increasingly utilised in the treatment of osteochondral defects [33].

Angiotensin receptor activity is elevated in hypertrophic cartilage, contributing to the pathogenesis of osteoarthritis [32]. Angiotensin II stimulates transforming growth factor‐ß (TGF‐ß), which serves as the primary mediator responsible for fibrosis [5, 6]. Intra‐articular injection of TGF‐ß into the knees of adult rats has been shown to induce osteoarthritis [14]. Additionally, in another study, the removal of the TGF‐ß2 receptor in mature mice was found to protect the knee joint from developing osteoarthritis. Furthermore, the same study demonstrated that oral administration of losartan, an angiotensin II receptor blocker, prevented osteoarthritis development without the need to remove the TGF‐ß2 receptor [7]. Losartan, commonly used as an antihypertensive agent, functions as an angiotensin receptor blocker and is typically administered orally. In a study investigating the effects of losartan on damaged cartilage, oral administration of losartan was shown to promote the formation of hyaline‐like cartilage [30]. However, concerns have been raised due to the requirement for high oral doses and the associated systemic side effects, with hypotension, hyperkalemia and renal function impairment being the most common. In contrast, a recent study demonstrated that intra‐articular injection of losartan promoted cartilage regeneration with hyaline‐like characteristics in a rabbit model of microfracture surgery. The effect was dose‐dependent, and 1 mg per joint was identified as the most effective dose without signs of inflammation or toxicity [17].

The purpose of this study was to evaluate and compare the radiological, macroscopic, biomechanical and histological results of OAT and intralesional scaffolding combined with intra‐articular losartan administration in a rabbit model of osteochondral injury. The hypothesis of this study was that treatment of osteochondral defects in rabbit knees using scaffolding and intra‐articular losartan injection would produce results similar to those obtained with the osteochondral autograft technique.

## METHODS

### Study design

Ethical approval for this study was granted by the local institutional ethics committee (IRB No: E.77285). A total of 12 female New Zealand rabbits, each aged 6 months and weighing between 3 and 4 kg, were procured from the university's laboratory breeding facilities. Both the right and left hind knees of each rabbit were included in the study, with randomisation determining the treatment allocation for each limb. One knee was assigned to receive the scaffold combined with intra‐articular losartan injection (Group A), while the contralateral knee was treated with osteochondral autograft transplantation (Group B) (*n* = 6).

### 3D bioprinted scaffold preparation

A total of 10 mL of solution is made from the mixed solution containing 4% alginate, 5% gelatin B and 1% hyaluronic acid. The solution is stirred for 2 h at 37°C. Drop 0.9 mL of 2% CaCl_2_ solution and wait for half an hour for the gel to cross‐link. The prepared solution is loaded into the syringe. The syringe is inserted into the 3D printer. During printing, 15% CaCl_2_ solution is sprayed on the scaffold once in each layer. After the scaffold printing is completed, 20% CaCl_2_ solution is sprayed seven times on the scaffold at 10‐min intervals. The scaffold, which is kept at +4°C for 1 day, is prepared with 95% ethanol and 33 mM EDC‐15 mM NHS solution the next day. The scaffold is immersed in 15 mL of the prepared solution. For the crosslinking process, the scaffold is kept in the solution at 37°C for 24 h. The scaffold taken from the oven (37°C) is removed from the crosslinking solution and the sterilisation processes are as follows: 10 min distilled water, 30 min pure ethanol 15 min phosphate‐buffered saline (PBS) (pH 7.4).

### Surgical procedure

**Figure 1:**
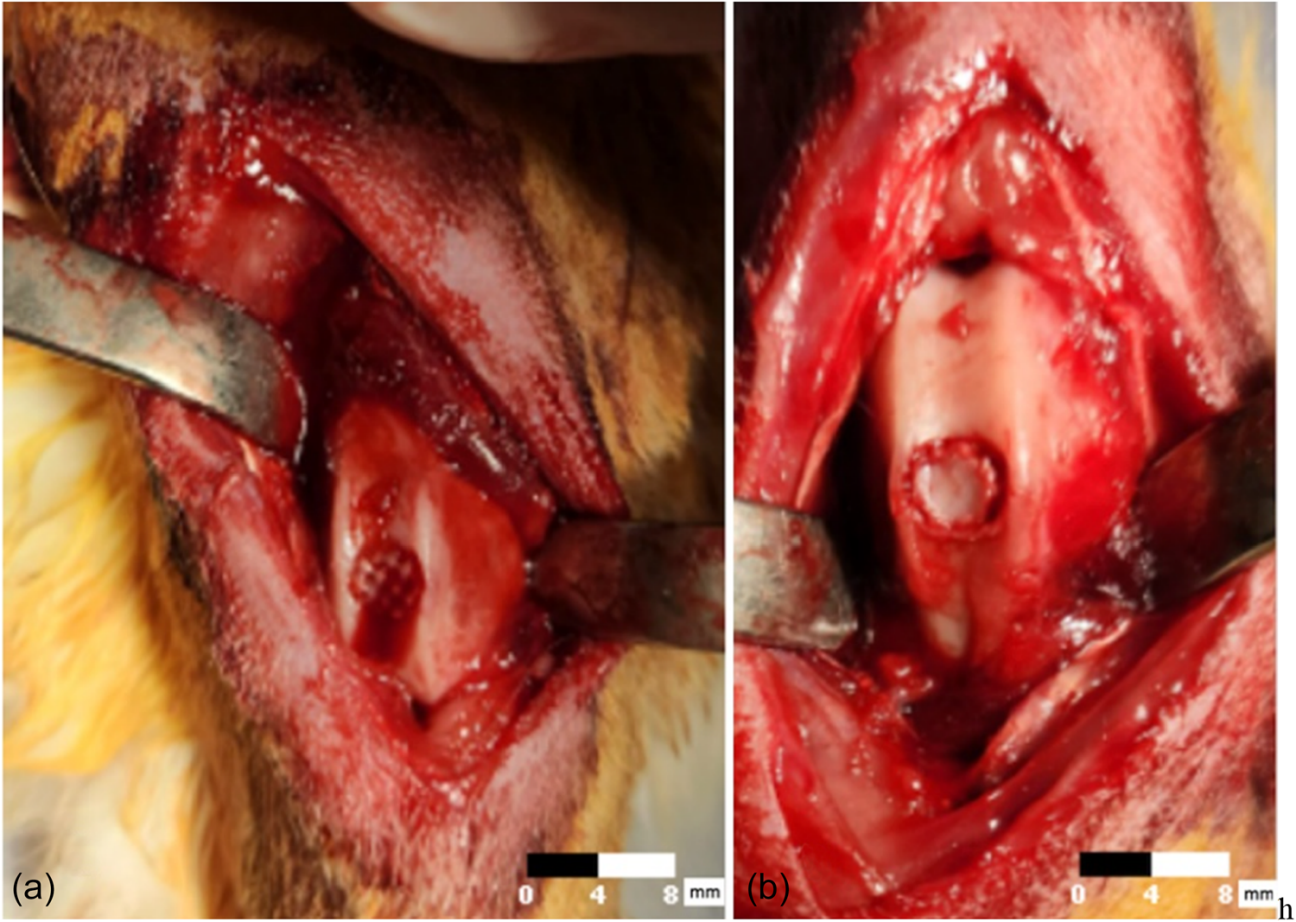
Images of surgical procedures. (a) Creation of a 4 mm wide and 4 mm deep full‐thickness osteochondral defect in the patellofemoral groove and repair of the created osteochondral defect with a three‐dimensional bioprinted scaffold. (b) Creation of a 4 mm wide and 4 mm deep full‐thickness osteochondral defect in the patellofemoral groove and repair of the created osteochondral defect with the same autograft.

Anaesthesia was induced via the marginal ear vein using xylazine (5 mg/kg, IV) and ketamine (50 mg/kg, IV), followed by the intravenous administration of prophylactic cefazolin (40 mg/kg). The hind limbs of each animal were shaved, prepared with a sterile antiseptic solution and draped in a sterile manner. The knee joint was identified by palpation and accessed via a midline incision. After incising the skin and subcutaneous tissue, a medial parapatellar approach was employed, with the incision extending medially to the patella. The patella was subsequently dislocated laterally to expose the patellofemoral groove. A full‐thickness osteochondral defect measuring 4 mm in width and 4 mm in depth was created in the trochlear region using a mosaicplasty instrument set. In Group A, the defect was filled with a scaffold (Figure 1a). After ensuring that the scaffold was flush with the surrounding healthy cartilage, the patella was reduced and the scaffold was appropriately covered. The extensor mechanism was then repaired. The subcutaneous tissue and skin were sutured in layers. A losartan solution (10 mg/mL) was prepared by dissolving 10 mg of losartan in 1 mL of PBS and sterilised through a 0.22 μm syringe filter. Then, 0.1 mL of the solution (equivalent to 1 mg losartan) was intra‐articularly injected into the knee joint.

In Group B, the osteochondral defect was repaired by reimplanting the osteochondral fragment that had been previously removed, as described in prior studies [15, 16] (Figure 1b). The patella was repositioned, and the osteochondral fragment was secured in place. The extensor mechanism was repaired, followed by closure of the subcutaneous tissue and skin. Postoperative dressing was applied. Animals received continued cefazolin prophylaxis for 24 h postoperatively and were allowed free movement in their cages with unrestricted access to food and water. In Group A, intra‐articular injections of 1 mg losartan (1 mL solution) were administered via a lateral parapatellar approach at 2 and 4 weeks postoperatively. All animals were followed for a total of 6 months.

### Follow‐up

During the postoperative follow‐up period, one rabbit from Group B, in which the osteochondral autograft was applied, developed an infection in the left knee, along with a wound site complication. Despite irrigation and debridement, the infection persisted, and this knee was subsequently excluded from the study. Additionally, one rabbit was entirely excluded from the study due to a femoral fracture sustained during the hammering process while removing the osteochondral autograft. Furthermore, two rabbits succumbed to complications related to postoperative anaesthesia. As a result, a total of nine animals were followed until the study's conclusion at 6 months. The final analysis included nine knees in Group A and eight knees in Group B.

### Radiological evaluation

Bilateral knee magnetic resonance imaging (MRI) was performed on each anaesthetised animal before sacrifice. A 3 Tesla Siemens screw XQ MRI device was used. (Coil: Knee Coil 18 channels Siemens). Thin sections of 2 mm were taken. Samples were acquired with a repetition time of 35 ms, an echo time of 19 ms (effective), a field of view (Fov) of 121 × 148, and an acquisition time (TA) of 3.54. A personalised knee protocol was applied to all samples using a cartilage‐sensitive fast spin echo sequence in the sagittal plane. The MRI evaluation was performed by a single‐blinded radiologist.

### Biomechanical evaluation

**Figure 2:**
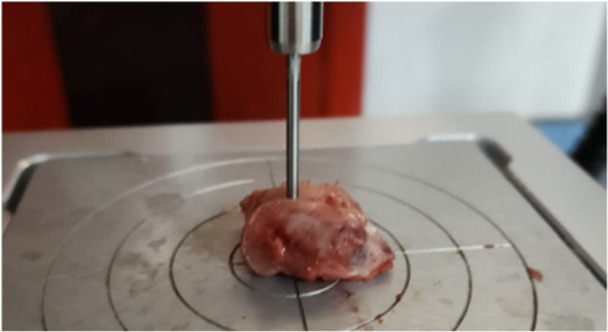
Biomechanical loading test with Stable Micro Systems.

The samples were placed in a phosphate‐based solution and kept at room temperature for 3 h. For biomechanical testing, the samples were positioned on the metal surface, and the cylindrical indentation (2 mm in diameter) was applied perpendicularly to the upper half of the defect area. The samples were kept moist throughout the experiment. A texture analyser called Stable Microsystems (Instron Model 3365, Instron Engineering Corporation) was used. A compression test was applied. Stress–strain graphs were drawn, and the load at which the cartilage and subchondral bone broke under continuous compression and the strain values at this value were compared (Figure 2).

### Histological evaluation

The histological analysis of the remaining intact lower half of the defect was initiated after the biomechanical testing. The extracted tissues were fixed in 10% buffered formaldehyde for at least 24 h in 10 times its volume. Each numbered tissue was methodically dissected. The samples were followed up for 12 h in an automatic tissue device. Then they were turned into paraffin blocks. 3–4 micron thick slide sections were taken from the paraffin blocks. Following deparaffinization, routine hematoxylin and eosin and Safranin‐0 staining were applied. Each slide was covered with a coverslip using entellan. Histological evaluation of the groups and Modified Wakitani Histological Scoring were performed.

### Statistical analysis

Number Cruncher Statistical System (NCSS) 2007 (Kaysville) program was used for statistical analysis. In addition to descriptive statistical methods (mean, standard deviation, median, frequency, ratio, minimum and maximum) in evaluating the study data, the Independent sample *t*‐test was used in two‐group comparisons. Pearson chi‐square test was used in comparing qualitative data. Significance was evaluated at *p* < 0.01 and *p* < 0.05 levels. In the preoperative period, power analysis and sample size calculation were performed using G*Power version 3.1.9.2 (Heinrich‐Heine‐Universität Düsseldorf). Based on an effect size (d) of 1.666, an alpha level of 0.05, and a power (1‐β) of 0.80, the required sample size was calculated to be six subjects per group [33].

## RESULTS

### Macroscopic evaluation

**Figure 3:**
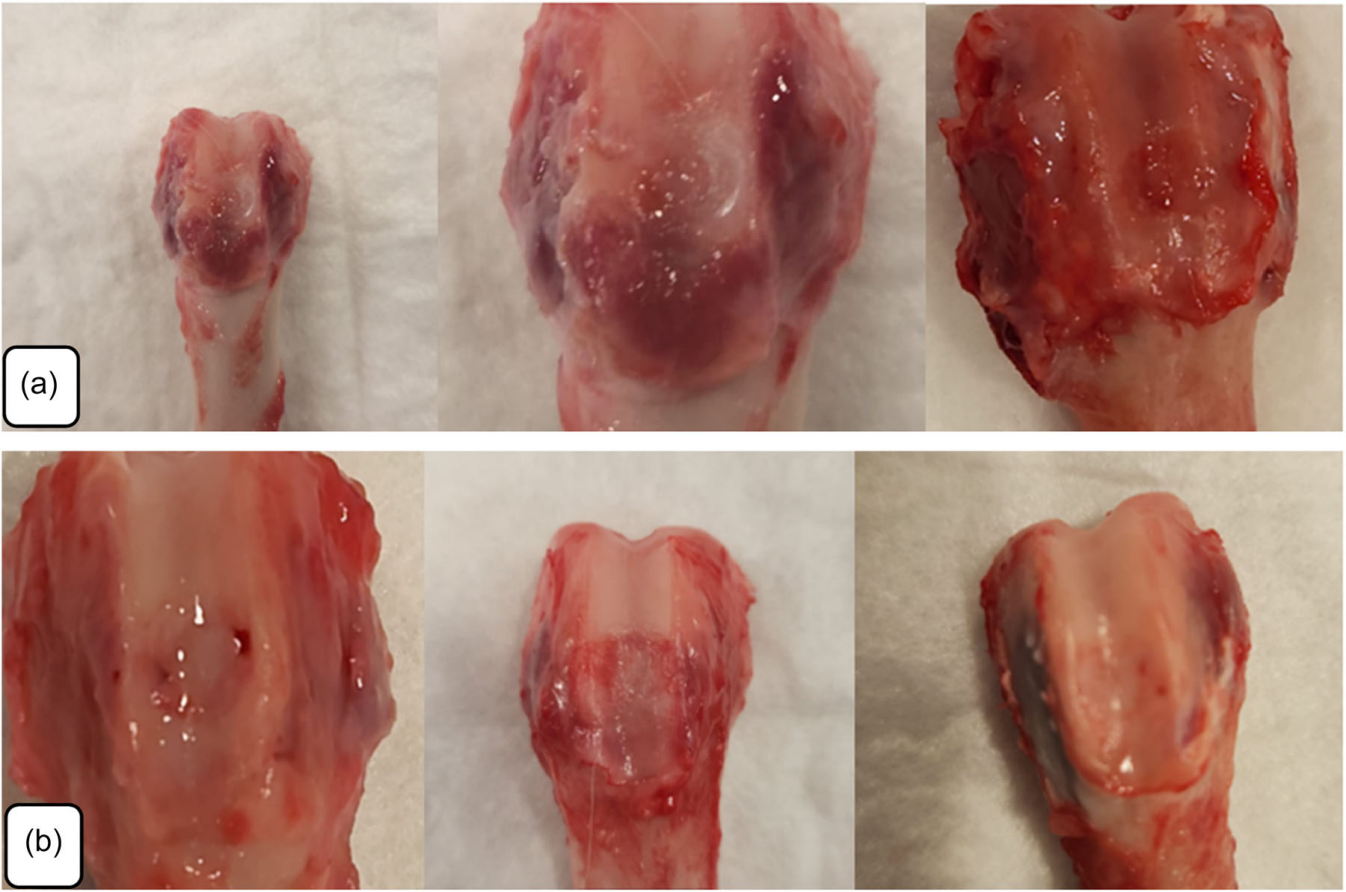
Images of macroscopic evaluation. (a) In Group A, the defect had well‐defined edges with no gaps, and the cartilage formed was at the same level as the healthy surrounding cartilage. (b) In Group B, the defective area fully integrated with the surrounding natural cartilage, and the surface properties aligned well with the neighbouring cartilage.

In all subjects from Group A and Group B, the defects had completely healed. In Group A, the defects were fully closed. The newly formed tissue appeared to be integrated into the defect area; however, the margins remained distinguishable due to tissue contrast or remodelling characteristics. No gap was observed between the regenerated tissue and the surrounding native cartilage. The regenerated cartilage within the defect area was found to be at the same level as the adjacent healthy cartilage (Figure 3a). On the other hand, in Group B, the margins between the regenerated and surrounding native cartilage appeared less distinct. The integration between the regenerated cartilage and the surrounding tissue was complete. The surface characteristics, including colour and texture, demonstrated a higher degree of conformity with the adjacent cartilage in Group B (Figure 3b).

### Radiological evaluation (MRI)

**Figure 4:**
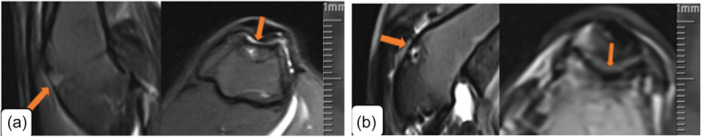
The MRI images of Group A and Group B. (a) T2‐weighted sagittal and axial MR images of Group A (3D‐printed scaffold + losartan). The arrows indicate the repair tissue filling the defect area, demonstrating smooth cartilage surfaces and continuity with the adjacent cartilage. Isointense signal characteristics are observed, and hyperintense regions beneath the scaffold may reflect subchondral bone remodelling. (b) T2‐weighted sagittal and axial MR images of Group B (osteochondral autograft). The arrows highlight the interface between the graft and native cartilage, showing surface regularity and isointense signal properties. Hyperintense zones below the graft suggest ongoing subchondral remodelling. Comparable MRI findings were noted in both groups, indicating successful integration and similar tissue response.

In both groups, the defect areas had closed, and the continuity of the subchondral bone and cartilage appeared natural (Figure 4). The cartilage surfaces were smooth, and the signal intensity of the grafts was isointense. In both groups, hyperintense signal areas of varying sizes were observed under the grafts, particularly at the contact points with the medullary bone.

### Biomechanical results

Biomechanically, there was no significant difference in the maximum stress values during loading between the two groups (*p* = 0.088). Similarly, the maximum strain values under load showed no statistically significant difference across the groups (*p* = 0.847) (Table 1).

**Table 1:** Evaluation of biomechanical measurement results of both groups.

	Group A		Group B		*p* value^a^
	Mean ± SD	Min–Max	Mean ± SD	Min–Max	
Max‐load stress (MPa)	68.33 ± 35.00	17.69–120.20	62.31 ± 32.11	10.55–102.10	0.579
Max‐load strain (%)	43.15 ± 10.96	35.76–64.30	41.22 ± 13.59	26.40–68.70	0.731

^a^ Mann–Whitney *U*‐test.

### Histopathological results

**Figure 5:**
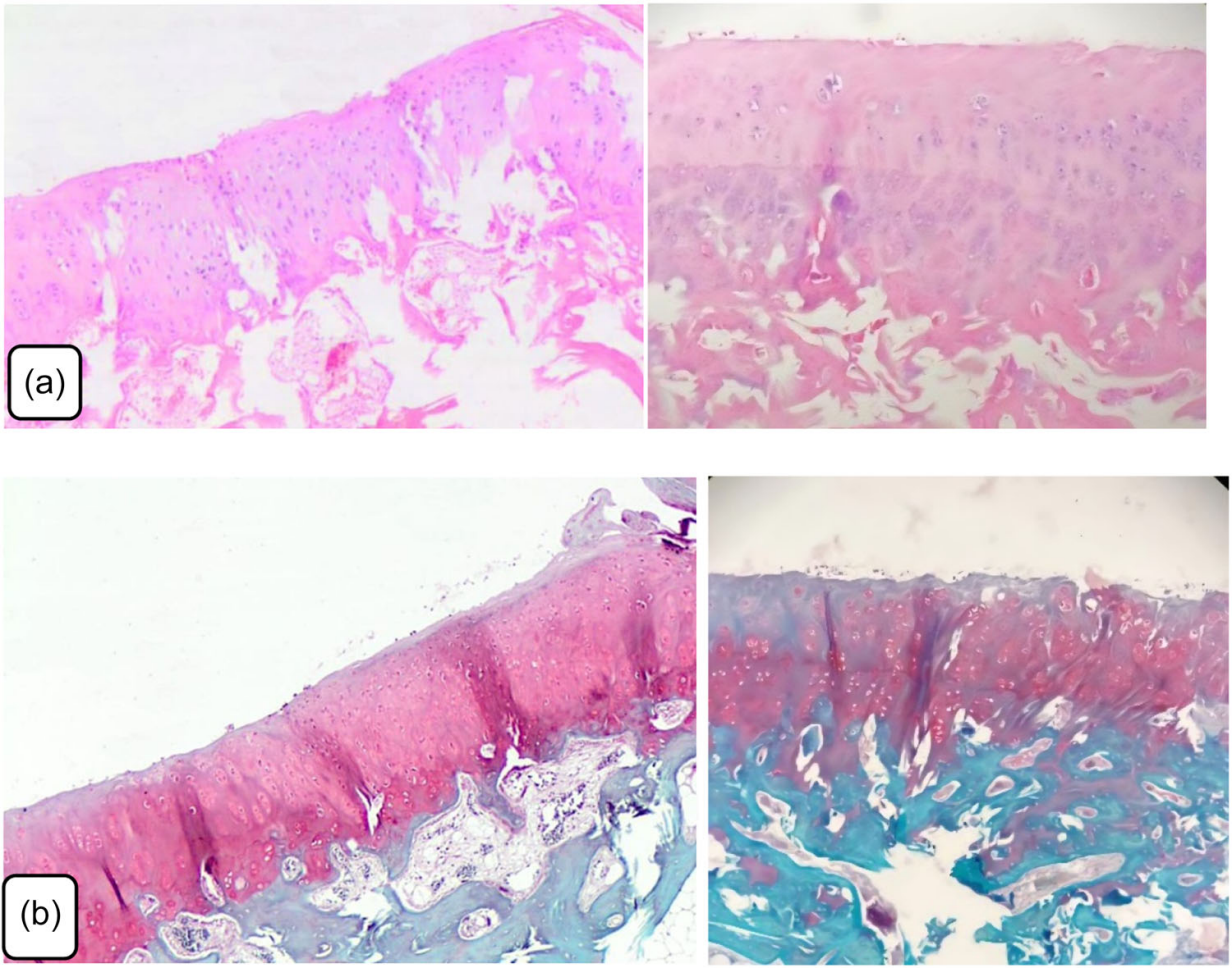
Histopathological examination of articular cartilage of osteochondral defect repair in Group A using hematoxylin and eosin and safranin staining. (a) Group A osteochondral defect healing area and surrounding tissue, near complete healing (hematoxylin and eosin, x40). (b) Group A osteochondral defect healing area and surrounding tissue, near complete healing (Safranin, x40).

**Figure 6:**
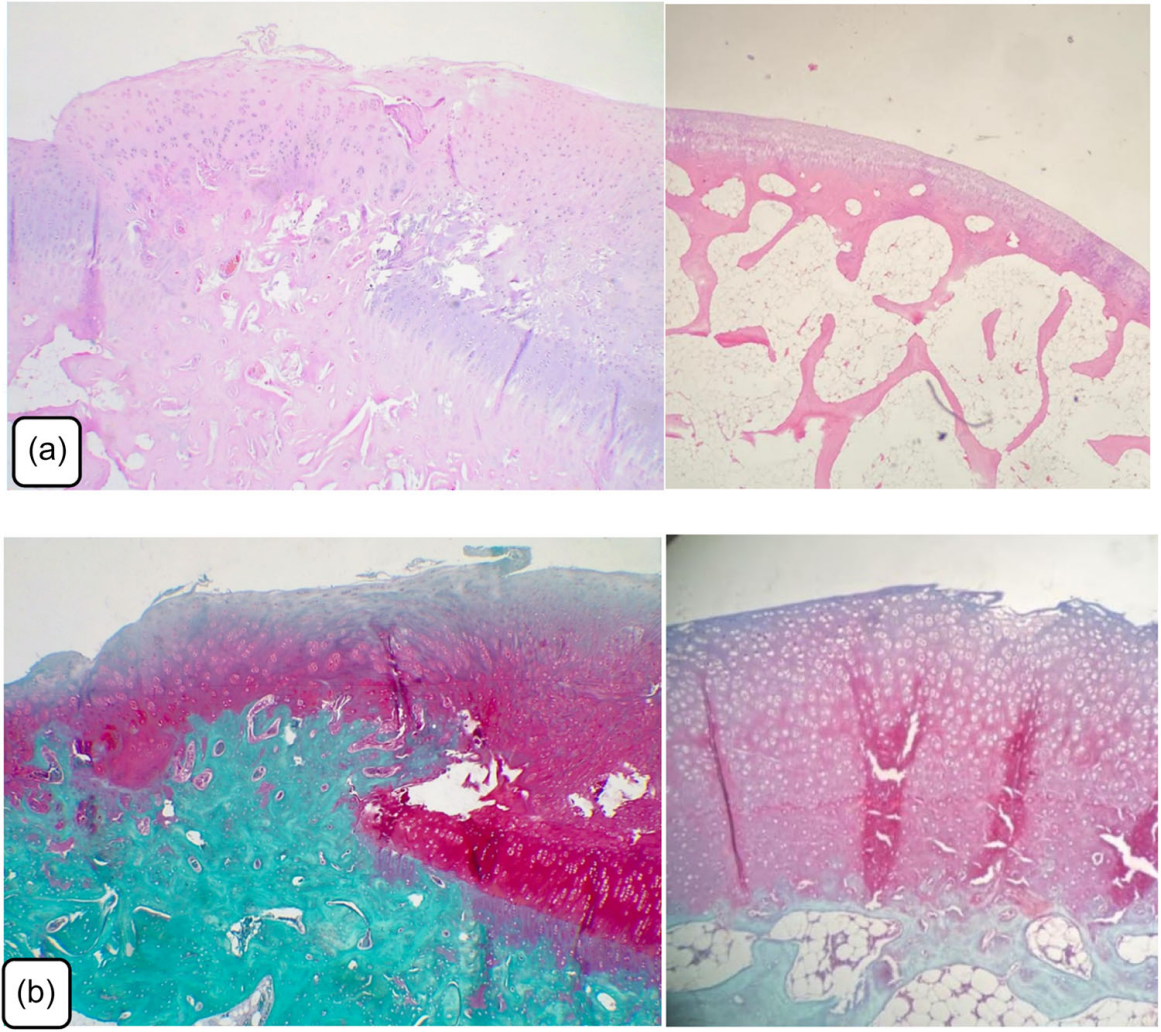
Histopathological examination of articular cartilage of osteochondral defect repair in Group B using hematoxylin and eosin and safranin staining. (a) Group B osteochondral defect healing area and surrounding tissue, near complete healing (hematoxylin and eosin, x40). (b) Osteochondral defect healing area and surrounding tissue, near complete healing (Safranin, x40).

Histopathological examination was performed using the modified Wakitani scoring system after hematoxylin‐eosin and Safranin‐O staining of the samples. Wakitani scoring system is a semiquantitative histological grading method used to evaluate cartilage repair. It assesses parameters such as cell morphology, matrix staining, surface regularity, cartilage thickness and integration with adjacent tissue. No significant difference was observed between the two groups in terms of cell morphology (*p* = 0.511*)*, matrix staining with Safranin‐O (*p* = 0.248), cartilage surface architecture (*p* = 0.223); cartilage thickness (*p* = 0.614), integration with the adjacent host cartilage (*p* = 0.614), and total Wakitani scores (*p* = 0.999) (Tables 2 and 3) (Figures 5 and 6).

**Table 2:** Evaluation of histological modified Wakitani score measurements according to groups.

		Group‐A	Group‐B	*p* value^a^
Cell morphology	Mostly cartilage	2	3	0.511
	Mostly hyaline cartilage	3	4	
	Mostly fibrocartilage	3	1	
	Mostly noncartilage	0	0	
	Noncartilage	0	0	
Matrix staining	Normal	1	3	0.248
	Slightly reduced	7	5	
	Markedly reduced	0	0	
	No metachromatic stain	0	0	
Surface regularity	Smooth (>3/4)	1	3	0.223
	Moderate (>1/2–3/4)	5	5	
	Irregular (1/4–2/1)	2	0	
	Severely irregular (<1/4)	0	0	
Cartilage thickness	>2/3	5	4	0.614
	1/3–2/3	3	4	
	<1/3	0	0	
Integration of implant with adjacent host cartilage	Both edges integrated	7	7	0.999
	One edge integrated	1	1	
	Neither edge integrated	0	0	

^a^ Pearson chi‐square.

**Table 3:** Evaluation of histological results of both groups according to modified Wakitani total score.

Group‐A			Group‐B		
	Mean ± SD	Min–Max (median)	Mean ± SD	Min–Max (median)	*p* value^a^
Wakitani total score	3.63 ± 2.13	1–6 (3)	2.63 ± 2.01	0–5 (3.5)	0.423

^a^ Mann–Whitney *U*‐test.

## DISCUSSION

The main finding of this study was that scaffold‐based approaches, in combination with intra‐articular losartan, may hold promising potential for osteochondral repair, offering a less invasive yet equally effective alternative to OAT. This introduces a new frontier in scaffold‐based acellular therapies, providing a future perspective for cartilage regeneration through the use of losartan as an antifibrotic agent to enhance endogenous scaffold‐mediated repair of osteochondral defects. Histological analysis revealed no significant differences in total Wakitani scores between the groups, suggesting that the scaffold–losartan combination can achieve tissue regeneration outcomes comparable to those of autograft techniques. Biomechanically, stress and strain values under maximum load were similar in both groups, highlighting the structural integrity of the repaired tissue obtained via the scaffold–losartan approach. MRI and macroscopic evaluations also showed no notable differences between groups, further supporting the comparable efficacy of losartan combined with an acellular scaffold for cartilage repair. It seems that a new star is rising on the horizon of orthobiologic treatments.

In addition to techniques such as microfracture, osteochondral autograft, autologous chondrocyte implantation and matrix‐associated autologous chondrocyte implantation, researchers have recently focused on scaffold‐based implants. Although hyaline‐like cartilage formation has been reported in animal studies in the literature, some clinical studies and secondary arthroscopies have reported the formation of both hyaline and fibrous cartilage [3]. Osteochondral autograft is a single‐stage procedure that promotes biological healing, and it has been reported to provide superior long‐term results compared to the microfracture technique [27, 28]. This is because healing is achieved with hyaline cartilage [25].

Losartan is an FDA‐approved antihypertensive drug that exerts antifibrotic effects by blocking angiotensin II receptors within the renin‐angiotensin‐aldosterone system. This effect is achieved by blocking the angiotensin II receptors located in the vascular walls. These receptors are also found in nonvascular tissues such as joint cartilage and synovial tissue [29, 31]. After angiotensin II binds to its receptor, the TGF‐ß signalling pathway is activated on the cell membrane. TGF‐ß is the mediator responsible for fibrosis [6, 21]. In an animal study, after gastrocnemius laceration, an oral angiotensin receptor blocker was administered to block the TGF‐ß signalling pathway during tissue healing. It was shown that fibrosis was reduced in this group [2]. TGF‐ß stimulates chondrocytes to increase collagen and proteoglycan synthesis [24], but it also causes fibrosis in synovial and other tissues [5, 18]. As a net effect, it disrupts joint homoeostasis, leading to osteoarthritis. Intra‐articular TGF‐ß injections in animal models have resulted in the development of osteoarthritis [14]. It has been reported that excessive TGF‐ß expression in the mouse knee leads to osteoarthritis and the formation of chondro‐osteophytes in synovial tissues [1]. Similar results have been found in studies involving intra‐articular TGF‐β injections in humans [13]. It has been demonstrated that oral losartan protects the joint from osteoarthritis in mice with meniscal instability [7]. Building on the hypothesis that losartan could potentially inhibit the fibrotic effects of TGF‐ß, oral losartan was administered to rabbits with osteochondral defects, resulting in reduced fibrosis and the formation of hyaline‐like cartilage [30]. To avoid the systemic side effects of oral losartan, the safe and effective intra‐articular dose of losartan was tested in rabbit knees and determined to be 1 mg [17]. This study aimed to explore an alternative treatment to osteochondral autograft for large osteochondral lesions. Histological assessments and Wakitani scores showed results similar to those of osteochondral autograft. In biomechanical analyses, no statistically significant differences were found. The similar results in biomechanical analyses support the hypothesis that no fibrin tissue formation occurred in the losartan group. Although there was no statistical difference, the autograft group was observed to perform slightly better. Yang et al. also reported similar findings after a 6‐month follow‐up, where a 3D bioprinted integrated osteochondral scaffold was used to repair cartilage defects in rabbit knees [33]. While the scaffold group demonstrated satisfactory cartilage regeneration, the autograft group showed slightly superior biomechanical and structural integration, likely due to the bone‐to‐bone healing advantage provided by native osteochondral plugs.

Our study employs the combination of 3D‐printed scaffolds and intra‐articular losartan injection as a technique that has not previously been investigated. This technique aims to facilitate the healing of osteochondral defects and promote the formation of hyaline‐like cartilage. Our histological findings support the formation of hyaline‐like cartilage, consistent with previous studies demonstrating that intra‐articular losartan at 1 mg/mL enhances microfracture‐mediated cartilage repair and promotes hyaline‐like regeneration in rabbit models [17]. Additionally, the biomechanical evaluations also yielded results that are in line with the findings in the literature [33]. Furthermore, similar histological and biomechanical results were obtained compared to OAT. The combination of 3D‐printed scaffolds and losartan offers several advantages: it is a less invasive approach that does not require graft harvesting, it is acellular and relies on endogenous repair through mesenchymal stem cell (MSC) mobilisation from bone marrow and synovial tissue, along with peripheral chondrocyte migration. Moreover, due to its easy integration with various scaffold types, it holds the potential to serve as a clinically applicable and readily available (off‐the‐shelf) therapeutic option. Previous studies have demonstrated that biological agents such as granulocyte colony‐stimulating factor (G‐CSF) can enhance MSC mobilisation; therefore, G‐CSF could potentially be integrated into this treatment model to further promote regeneration [19, 20].

This study has several limitations that should be considered when interpreting the results. First, the levels of losartan in blood and urine were not measured, nor were postoperative vital signs, such as blood pressure, monitored, limiting the assessment of potential systemic side effects. Additionally, there are inherent differences between rabbit and human cartilage in terms of structure and healing capacity, which may affect the translatability of these findings to clinical practice. Another limitation is the use of both knees for intervention in the same animal. In clinical practice, only one knee is typically treated, and bilateral procedures may impair normal postoperative recovery due to functional limitations. The follow‐up period in this study was relatively short, potentially restricting insights into the long‐term effects on cartilage repair. Furthermore, the use of a small sample size, common in animal studies, may limit the generalisability of the results. It is also noteworthy that the OAT procedure in this study was a reimplantation of the same osteochondral plug, rather than a true autograft harvested from a separate donor site. As a result, there was no donor site morbidity in Group B, which may have introduced a positive bias toward better healing in this group. However, this design choice also established a consistent baseline between groups by eliminating donor site complications in both, thereby improving the balance for comparative analysis. Finally, the lack of a control group to isolate and evaluate the specific effects of losartan alone constitutes an important limitation.

## CONCLUSION

This study highlights a groundbreaking therapeutic concept merging 3D‐printed intralesional scaffolding with intra‐articular losartan injections that may redefine the future of osteochondral repair. Offering a less invasive, acellular, and off‐the‐shelf solution, this innovative approach not only matches the biomechanical and histological outcomes of autograft techniques, but also opens a new era in cartilage regeneration. With its ability to harness endogenous repair mechanisms and its potential adaptability to diverse clinical scenarios, this strategy shines as a transformative and promising frontier in orthobiologic treatment.

## AUTHOR CONTRIBUTIONS

**Mesut Ok**: Design; drafting/writing. **Mahmud Aydin**: Design; data collection/processing; analysis/interpretation; literature review; drafting/writing; critical review. **Serkan Surucu**: Analysis/interpretation; critical review. **Halide Nur Urer**: Data collection/processing. **Mehmet Halis Cerci**: Idea/concept; data collection/processing; drafting/writing. **Murat Yilmaz**: Literature review. **Mesut Sonmez**: Literature review. **Mahir Mahirogullari**: Idea/concept; critical review.

## CONFLICT OF INTEREST STATEMENT

The authors declare no conflicts of interest.

## ETHICS STATEMENT

Ethical approval for this study was granted by the local institutional ethics committee (IRB No: E.77285).

## Data Availability

Encourages data sharing.

## References

[1] Bakker AC , Van de Loo FAJ , Van Beuningen HM , Sime P , van Lent PLEM , van der Kraan PM , et al. Overexpression of active TGF‐beta‐1 in the murine knee joint: evidence for synovial‐layer‐dependent chondro‐osteophyte formation. Osteoarthr Cartil. 2001;9(2):128–136. 10.1053/joca.2000.0368 11237660

[2] Bedair HS , Karthikeyan T , Quintero A , Li Y , Huard J . Angiotensin II receptor blockade administered after injury improves muscle regeneration and decreases fibrosis in normal skeletal muscle. Am J Sports Med. 2008;36(8):1548–1554. 18550776 10.1177/0363546508315470

[3] Benthien JP , Behrens P . Nanofractured autologous matrix induced chondrogenesis (NAMIC©)—Further development of collagen membrane aided chondrogenesis combined with subchondral needling. Knee. 2015;22(5):411–415. 26190333 10.1016/j.knee.2015.06.010

[4] Berruto M , Ferrua P , Uboldi F , Pasqualotto S , Ferrara F , Carimati G , et al. Can a biomimetic osteochondral scaffold be a reliable alternative to prosthetic surgery in treating late‐stage SPONK? Knee. 2016;23(6):936–941. 27592357 10.1016/j.knee.2016.08.002

[5] Bowen T , Jenkins RH , Fraser DJ . MicroRNAs, transforming growth factor beta‐1, and tissue fibrosis. J Pathol. 2013;229(2):274–285. 23042530 10.1002/path.4119

[6] Branton MH , Kopp JB . TGF‐β and fibrosis. Microb Infect. 1999;1(15):1349–1365. 10.1016/s1286-4579(99)00250-6 10611762

[7] Chen R , Mian M , Fu M , Zhao JY , Yang L , Li Y , et al. Attenuation of the progression of articular cartilage degeneration by inhibition of TGF‐β1 signaling in a mouse model of osteoarthritis. Am J Pathol. 2015;185(11):2875–2885. 26355014 10.1016/j.ajpath.2015.07.003 PMC4630169

[8] Eslaminejad MB . Mesenchymal stem cells as a potent cell source for articular cartilage regeneration. World J Stem Cells. 2014;6(3):344–354. 25126383 10.4252/wjsc.v6.i3.344 PMC4131275

[9] Gobbi A , Karnatzikos G , Kumar A . Long‐term results after microfracture treatment for full‐thickness knee chondral lesions in athletes. Knee Surg Sports Traumatol Arthrosc. 2014;22(9):1986–1996. 24051505 10.1007/s00167-013-2676-8

[10] Gomoll AH , Minas T . The quality of healing: articular cartilage. Wound Repair Regen. 2014;22(Suppl 1):30–38. 24813362 10.1111/wrr.12166

[11] Goyal D . Recent advances and future trends in articular cartilage repair. J Arthrosc Surg Sports Med. 2020;1:159–173.

[12] Gudas R , Gudaitė A , Pocius A , Gudienė A , Čekanauskas E , Monastyreckienė E , et al. Ten‐year follow‐up of a prospective, randomized clinical study of mosaic osteochondral autologous transplantation versus microfracture for the treatment of osteochondral defects in the knee joint of athletes. Am J Sports Med. 2012;40(11):2499–2508. 23024150 10.1177/0363546512458763

[13] Guermazi A , Kalsi G , Niu J , Crema MD , Copeland RO , Orlando A , et al. Structural effects of intra‐articular TGF‐β1 in moderate to advanced knee osteoarthritis: MRI‐based assessment in a randomized controlled trial. BMC Musculoskelet Disord. 2017;18(1):461. 29145839 10.1186/s12891-017-1830-8 PMC5689208

[14] Itayem R , Mengarelli‐Widholm S , Hulth A , Reinholt FP . Ultrastructural studies on the effect of transforming growth factor‐β1 on rat articular cartilage. APMIS. 1997;105(3):221–228. 9137518 10.1111/j.1699-0463.1997.tb00562.x

[15] Jin LH , Choi BH , Kim YJ , Park SR , Jin CZ , Min BH . Implantation of bone marrow‐derived buffy coat can supplement bone marrow stimulation for articular cartilage repair. Osteoarthr Cartil. 2011;19(12):1440–1448. 10.1016/j.joca.2011.07.012 21843651

[16] Jing L , Zhang J , Leng H , Guo Q , Hu Y . Repair of articular cartilage defects in the knee with autologous iliac crest cartilage in a rabbit model. Knee Surg Sports Traumatol Arthrosc. 2015;23(4):1119–1127. 24573237 10.1007/s00167-014-2906-8

[17] Logan CA , Gao X , Utsunomiya H , Scibetta AC , Talwar M , Ravuri SK , et al. The beneficial effect of an intra‐articular injection of losartan on microfracture‐mediated cartilage repair is dose dependent. Am J Sports Med. 2021;49(9):2509–2521. 34259597 10.1177/03635465211008655

[18] Madsen SF , Madsen SS , Madrid AS , Andersen MR , Bay‐Jensen AC , Thudium CS . Fibrotic remodeling in joint diseases: induction and inhibition of fibrosis in fibroblast‐like synoviocytes. Transl Med Commun. 2024;9:18.

[19] Marmotti A , Bonasia DE , Bruzzone M , Rossi R , Castoldi F , Collo G , et al. Human cartilage fragments in a composite scaffold for single‐stage cartilage repair: an in vitro study of the chondrocyte migration and the influence of TGF‐β1 and G‐CSF. Knee Surg Sports Traumatol Arthrosc. 2013;21(8):1819–1833. 23143386 10.1007/s00167-012-2244-7

[20] Marmotti A , Castoldi F , Rossi R , Marenco S , Risso A , Ruella M , et al. Bone marrow‐derived cell mobilization by G‐CSF to enhance osseointegration of bone substitute in high tibial osteotomy. Knee Surg Sports Traumatol Arthrosc. 2013;21(1):237–248. 22872005 10.1007/s00167-012-2150-z

[21] Meng X , Nikolic‐Paterson DJ , Lan HY . TGF‐β: the master regulator of fibrosis. Nat Rev Nephrol. 2016;12(6):325–338. 27108839 10.1038/nrneph.2016.48

[22] Mithoefer K , McAdams T , Williams RJ , Kreuz PC , Mandelbaum BR . Clinical efficacy of the microfracture technique for articular cartilage repair in the knee: an evidence‐based systematic analysis. Am J Sports Med. 2009;37(10):2053–2063. 19251676 10.1177/0363546508328414

[23] Ow ZGW , Cheang HLX , Koh JH , Koh JZE , Lim KKL , Wang D , et al. Does the choice of acellular scaffold and augmentation with bone marrow aspirate concentrate affect short‐term outcomes in cartilage repair? a systematic review and meta‐analysis. Am J Sports Med. 2023;51(6):1622–1633. 35225004 10.1177/03635465211069565

[24] Pujol JP , Galera P , Redini F , Mauviel A , Loyau G . Role of cytokines in osteoarthritis: comparative effects of interleukin 1 and transforming growth factor‐beta on cultured rabbit articular chondrocytes. J Rheumatol Suppl. 1991;27:76–79. 2027137

[25] Rădulescu RA , Cirstoiu CF , Bădilă AE . Arthroscopical and histological study of cartilaginous lesions treated by mosaicplasty. J Med Life. 2010;3(4):407–411. 21254739 PMC3019066

[26] Shapiro F , Koide S , Glimcher MJ . Cell origin and differentiation in the repair of full‐thickness defects of articular cartilage. J Bone Jt Surg. 1993;75(4):532–553. 10.2106/00004623-199304000-00009 8478382

[27] Solheim E , Hegna J , Øyen J , Harlem T , Strand T . Results at 10 to 14 years after osteochondral autografting (mosaicplasty) in articular cartilage defects in the knee. Knee. 2013;20(4):287–290. 23482060 10.1016/j.knee.2013.01.001

[28] Solheim E , Hegna J , Strand T , Harlem T , Inderhaug E . Randomized study of long‐term (15‐17 years) outcome after microfracture versus mosaicplasty in knee articular cartilage defects. Am J Sports Med. 2018;46(4):826–831. 29253350 10.1177/0363546517745281

[29] Tang Y , Hu X , Lu X . Captopril, an angiotensin‐converting enzyme inhibitor, possesses chondroprotective efficacy in a rat model of osteoarthritis through suppression local renin‐angiotensin system. Int J Clin Exp Med. 2015;8(8):12584–12592. 26550169 PMC4612854

[30] Utsunomiya H , Gao X , Deng Z , Cheng H , Nakama G , Scibetta AC , et al. Biologically regulated marrow stimulation by blocking tgf‐β1 with losartan oral administration results in hyaline‐like cartilage repair: a rabbit osteochondral defect model. Am J Sports Med. 2020;48(4):974–984. 32027515 10.1177/0363546519898681

[31] Wang Y , Kou J , Zhang H , Wang C , Li H , Ren Y , et al. The renin‐angiotensin system in the synovium promotes periarticular osteopenia in a rat model of collagen‐induced arthritis. Int Immunopharmacol. 2018;65:550–558. 30412852 10.1016/j.intimp.2018.11.001

[32] Wu Y , Lu X , Li M , Zeng J , Zeng J , Shen B , et al. Renin‐angiotensin system in osteoarthritis: a new potential therapy. Int Immunopharmacol. 2019;75:105796. 31408841 10.1016/j.intimp.2019.105796

[33] Yang Y , Yang G , Song Y , Xu Y , Zhao S , Zhang W . 3D bioprinted integrated osteochondral scaffold‐mediated repair of articular cartilage defects in the rabbit knee. J Med Biol Eng. 2020;40:71–81.

